# Low HBV knowledge is associated with low HBV vaccination uptake in general adult population despite incentivization of HBV vaccination

**DOI:** 10.1186/s12879-024-09326-9

**Published:** 2024-05-03

**Authors:** Thanh Van Kim, Trang Ngoc Doan Pham, Brian Do, Diem Vu Bich Dao, Dan Xuan Nguyen, William Lee, Robert Gish, Gary Mize, Amy Trang, Anh Le, Loc Thi Bich Phan, Thi-Thuy-Dung Ngo, Hai Thanh Phan, Binh Tan Nguyen, Hong Kim Tang, Doan Y Dao

**Affiliations:** 1 Department of Epidemiology, Pham Ngoc Thach University of Medicine, Ho Chi Minh City, Viet Nam; 2 Viet Nam Viral Hepatitis Alliance, Reston, VA USA; 3 Department of Epidemiology and Biostatistics, School of Public Health, University of Illinois at Chicago, Chicago, IL USA; 4 University of California, La Jolla, San Diego, USA; 5 Department of Internal Medicine, UT Southwestern Medical Center, Dallas, TX USA; 6 Hepatitis B Foundation, Doylestown, PA USA; 7 Medic Medical Center, Ho Chi Minh City, Viet Nam; 8 Ho Chi Minh City Department of Health, Ho Chi Minh City, Viet Nam; 9 Center of Excellence for Liver Disease in Viet Nam, Ross Research Building, Room 908, Johns Hopkins University School of Medicine, 720 Rutland Avenue, Baltimore, MD 21205 USA

**Keywords:** Hepatitis B virus, Vaccination, Health knowledge, Incentivization, Adult, Viet Nam, Lower-middle-income country

## Abstract

**Background:**

Hepatitis B virus (HBV) vaccination in Vietnamese adults remains low and unequally distributed. We conducted a study on HBV-naïve adults living in Ho Chi Minh City, Viet Nam, to determine barriers associated with HBV vaccination uptake after removing the financial barrier by providing free coupons for HBV vaccination.

**Methods:**

After being screened for HBsAg, anti-HBs, and anti-HBc, 284 HBV-naïve study participants aged 18 and over (i.e., negative for HBsAg, anti-HBs, and anti-HBc total) were provided free 3-dose HBV vaccine coupons. Next, study participants’ receipt of 1st, 2nd, and 3rd doses of HBV vaccine was documented at a pre-specified study healthcare facility, where HBV vaccines were distributed at no cost to the participants. Upon study entry, participants answered questionnaires on sociodemographics, knowledge of HBV and HBV vaccination, and related social and behavioral factors. The proportions of three doses of HBV vaccine uptake and their confidence intervals were analyzed. Associations of HBV vaccine initiation with exposures at study entry were evaluated using modified Poisson regression.

**Results:**

98.9% (281 of 284) of study participants had complete data and were included in the analysis. The proportion of participants obtaining the 1st, 2nd, and 3rd doses of HBV vaccine was 11.7% (95% Confidence Interval [95% CI] 8.0-15.5%), 10.7% (95%CI 7.1–14.3%), and 8.9% (95%CI 5.6–12.2%), respectively. On the other hand, participants were more likely to initiate the 1st dose if they had adequate knowledge of transmission (adjusted relative risk [aRR] = 2.58, 95% CI 1.12–5.92), adequate knowledge of severity (aRR = 6.75, 95%CI 3.38–13.48), and annual health-checking seeking behavior (aRR = 2.04, 95%CI 1.07–3.87).

**Conclusion:**

We documented a low HBV vaccination uptake despite incentivization. However, increased vaccine initiation was associated with better HBV knowledge and annual health check-up adherence. When considering expanding HBV vaccination to the general adult population, we should appreciate that HBV knowledge is an independent predictor of vaccine uptake.

**Supplementary Information:**

The online version contains supplementary material available at 10.1186/s12879-024-09326-9.

## Background

 Hepatitis B virus (HBV) infection causes liver cirrhosis, liver failure, liver cancer, and death. Up to 40% of persons living with chronic HBV infection progress to liver cancer during their lifetime [1]. Globally, chronic HBV infection affects 296 million people and contributes to 820,000 deaths yearly [2]. The Western Pacific region is considered as an intermediate HBV-endemic region by World Health Organization (WHO), with an estimated prevalence of 7.1% (6.3–7.9%) [3]. Viet Nam, a lower-middle-income country (LMIC) in the Western Pacific region, has a population of 97 million and an estimated HBV prevalence of 7–8% [4]. Since 2018, HBV has been the most common etiology of liver cancer in Viet Nam [5, 6].

Childhood vaccination for HBV is cost-effective and the best strategy to prevent new HBV infections and thus liver cancer. The HBV vaccine is the first “anti-cancer” vaccine approved the United States (US) Food and Drug Administration because it prevents new HBV infection, thereby preventing liver cancer caused by HBV. In Viet Nam, the HBV vaccine was introduced to the national Expanded Program on Immunization (EPI) in 2003 and has significantly reduced HBV infections among infants born after the EPI program roll-out [7]. HBV vaccine coverage in infants was reported to achieve the WHO target in 2020, including 90% for three doses and 50% for birth doses in the country [8].

By contrast, adult HBV vaccination is only recommended in high-risk populations (e.g., IV drug users, health care workers, sex workers) by Viet Nam’s Ministry of Health [9]. As a result, in Viet Nam, the coverage for adult HBV vaccination is low for those born before the national EPI implementation for childhood HBV vaccination [10]. Approximately only 18.7% of Ho Chi Minh City (HCMC) adults (i.e., 18 years or older) had serological evidence of HBV vaccination, and 37.7% were susceptible to HBV infection [10]. Furthermore, the distribution of HBV vaccine coverage among the adults in HCMC was unequal by geographical areas, socioeconomic statuses, and HBV educational levels [10].

In 2016, the World Health Assembly passed the Global Health Sector Strategy on Viral Hepatitis, which consists of a 90% reduction in new infections as a significant target for HBV elimination [11]. In 2022, the US Centers for Disease Control and Prevention (CDC) expanded its recommendation for HBV vaccination to include all adults ages 19–59 as a step toward global HBV elimination [12].

To promote a national dialogue towards an expanded HBV vaccination policy, reduction of new HBV infections, and hepatitis B elimination in Viet Nam and potentially in other LMICs, we examined the barrier(s) to adulthood HBV vaccination through an incentive-driven program for unimmunized or under-immunized individuals. Specifically, we (1) assessed the proportions of HBV vaccine uptake among HBV-naïve persons after providing them with free coupons for a 3-shot HBV vaccine series, and (2) examined factors associated with HBV vaccine initiation rates in the same population.

## Materials and methods

### Study design and intervention

**Fig. 1:**
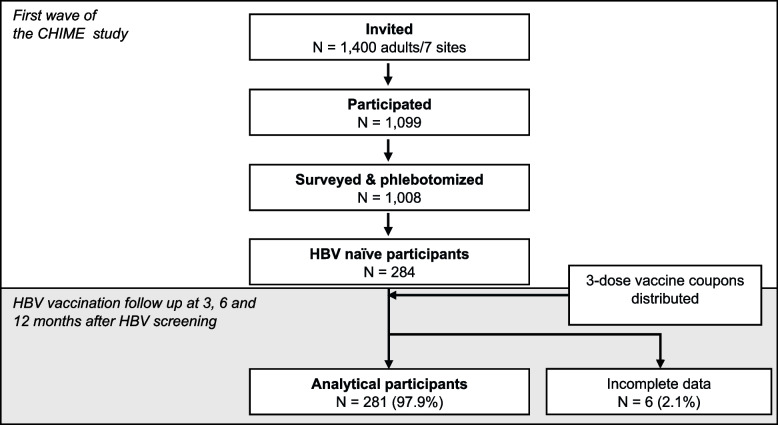
The participant flow and follow-up. Area in gray indicates this current study

This study belongs to the first wave of a comprehensive HBV screening and access-to-care program involving 20,000 adults representing the population of HCMC conducted during 2016–2020.(i.e., Conquering Hepatitis vIa Hepatitis Elimination or CHIME). The detailed CHIME study method was described elsewhere [13, 14]. In brief, from a multi-stage clustered study using the probability proportional to size approach, seven communes were selected from a total of 25 designated sites for the first wave. Two neighborhoods from each of the seven selected communes were randomly enrolled in a serosurvey. Invitations were sent to 200 adults for each commune. A total of 1,099 of the 1,400 invited participants (response rate of 78.5%) reported to the screening sites (i.e., commune’s health clinics), answered the Knowledge Attitude and Practice (KAP) questionnaires, and agreed to phlebotomy. Each participant was screened for HBV seromarkers using Roche Diagnostics’ Elecsys® HBsAg II, anti-HBs II, and anti-HBc II and analyzed using the Cobas® e 801 system [15–17]. Of 1,099 participants, 1,008 (91.7%) completed KAP questionnaires and phlebotomy. Two to four weeks after screening, the results were returned to participants in a sealed envelope. For the screening results, of 1,008 participants, 284 (28.2%) were HBV-naïve with HBsAg (-), anti-HBs Ab < 10mIU/mL, and anti-HBc total Ab (-), thus, susceptible to HBV infection. The difference in characteristics of this study’s interest between HBV naïve and other HBV status groups is presented in Appendix Table 1. The first wave was conducted between June 2016 to December 2017. The schematic participant flow is presented in Fig. 1.

**Fig. 2:**
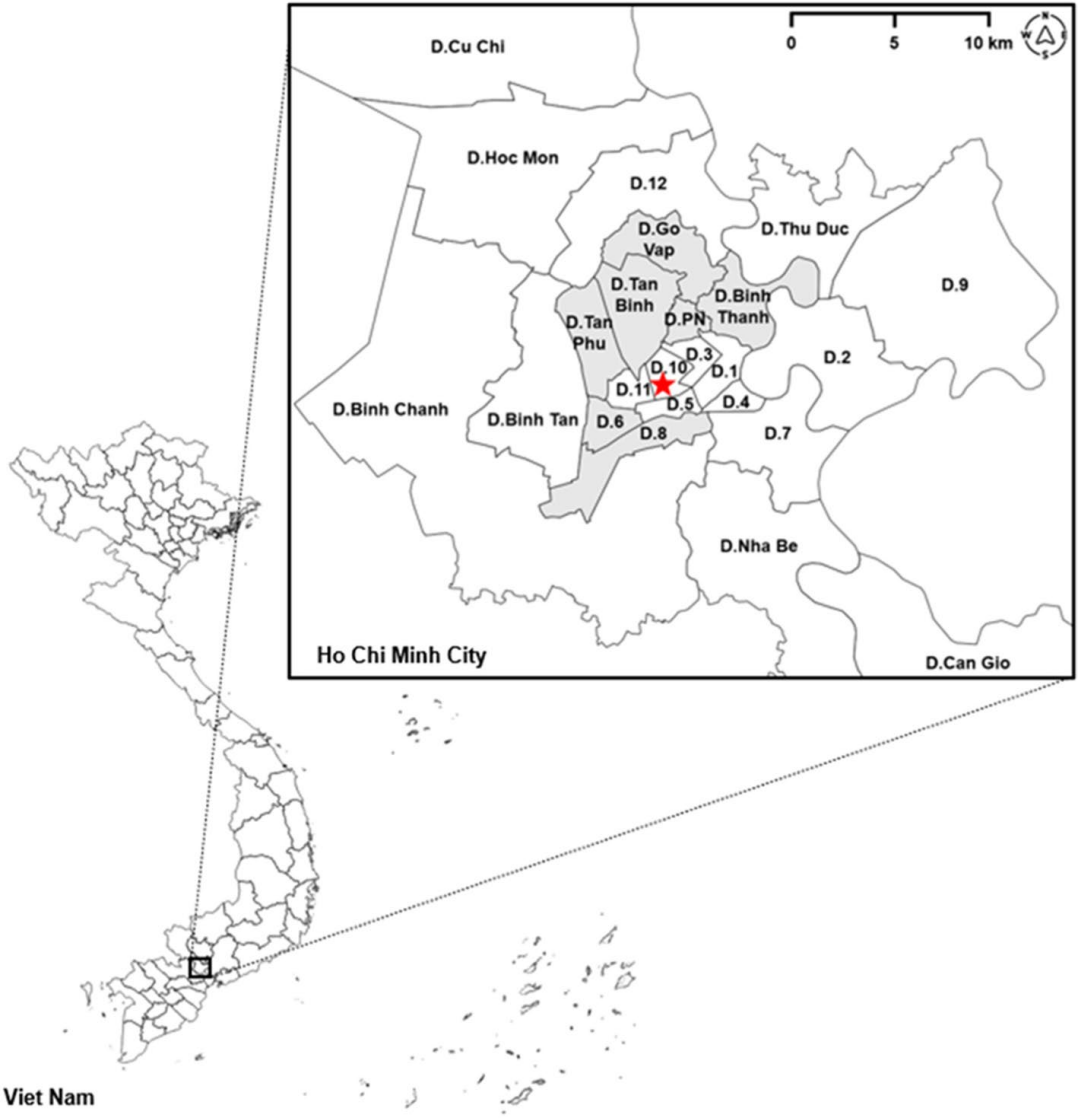
Map of Viet Nam and the zoomed-in Ho Chi Minh City. In Ho Chi Minh City, districts in slight gray were participants’ reported residence. District 10 is where Medic Medical Center is located and marked with a red star. Abbreviation: D.8 and D.Go Vap stand for District 8 and District Go Vap; D.PN stands for District Phu Nhuan

The 284 HBV naïve participants (HBsAg (-), anti-HBs Ab < 10mIU/mL, and anti-HBc total Ab (-)) were eligible to be recruited in this study. They received coupons for a free three-shot series of HBV vaccination at the Medic Medical Center (MMC). We followed up on vaccination coupon usage at MMC for up to one year after returning the screening results to participants. The MMC is located in District 10 – a central district in Ho Chi Minh City (Figure 2, Red Star). Engerix B® was the vaccine of choice to provide to all participants. We selected MMC because we have established a long-term working relationship with the medical center.

### Variable measurements

The primary outcome is HBV vaccination uptake, represented by the coupon used for the 1st, 2nd, or 3rd shot and recorded monthly by the study team using the MMC information system. The time to vaccine uptake was also documented. Participants were followed for up to one year after receiving the free vaccine coupons.

#### Demographics and socioeconomic statuses (SES)

Demographic information was collected via a questionnaire, including age, gender, ethnicity, marital status, educational attainment, area of residence, and income level. Age was regrouped as 18-30, 31-40, 41-50, and over 50 years old. Ethnic groups other than the Kinh, representing 95% of Vietnamese ethnicities, were labeled “Others.” Areas of residence were dichotomized based on their geographical distance to MMC into under 8 kilometers and 8 kilometers and above. We chose 8km as the average traveling distance from the participant’s residence to the MMC based on calculating the mean distance from the seven communes participating in this study to the MMC (Appendix Table 2).

#### Knowledge of HBV and HBV vaccination

The self-administered questionnaires developed on Vietnamese American populations with good construct validity [18] were adapted to document participants’ knowledge of HBV’s transmission routes, severity, and HBV vaccination. Participants’ responses to each question were assigned 1 point if correct (see the questionnaire and scoring system in the Appendix); otherwise, 0 points. Total scores were calculated based on 7 items for knowledge of transmission routes, 4 for knowledge of severity, and 4 for knowledge of vaccination. The face validity of the Vietnamese questionnaires was considered acceptable by the research team’s hepatologists, epidemiologists, social scientists, and some of the participants.

Knowledge of seven potential transmission routes of HBV was determined by the responses to: “Do you think that one can get viral hepatitis B by [provided behavior]?”. The provided behaviors included sharing a cigarette, sharing food or eating utensils, sharing a toothbrush, coughing or sneezing, sexual intercourse, sharing or using used needles, and childbirth. Knowledge of the severity of HBV was assessed with four questions: “Do you think patients with viral hepatitis B can [provided information]?” The provided information included being infected for life, developing liver cancer, and dying of HBV, cannot be treated. Knowledge of HBV vaccination was assessed with four questions: “Do you think HBV vaccine is/can [provided characteristics]?”. The provided characteristics included being effective in preventing HBV, causing adverse events, being safe, and knowing where to get HBV vaccination.

#### Social and behavioral factors

Participants reported to the questions “Have you had a health check-up in the past 12 months?”, “Have you ever got HBV vaccination?”, “Have your family members been infected with viral hepatitis?”. The options included Yes, No, and Don’t Know.

### Statistical methods

The proportions of vaccine uptake and their Wald-typed confidence intervals were estimated for the 1^st^, 2^nd^, and 3^rd^ doses. Also, the number of days from receiving coupons until the 1^st^, 2^nd^, and 3^rd^ doses was evaluated in medians and interquartile ranges. Bivariate differences were statistically tested with a t-test or rank sum test (between continuous variables) or Chi^2^ or Fisher’s exact test (between categorical variables) where applicable.

Using modified Poisson regression with sandwich estimation, we modelled factors associated with HBV vaccine initiation rates because the 1^st^-dose vaccination rates were > 10% [19]. The purposeful selection of potential confounders (i.e., demographic variables) for multiple adjustments were based on an a priori conceptual framework [20]. Model-wise deletion was used to handle missing data.

The significance level for all hypothesis testing is set at 0.05. The precision and power analysis were presented in the Appendix. All data analyses were done in the R program (v.4.2.2) and RStudio (v.2023.03.0); packages in use included “tidyverse” (v.1.3.2) for data wrangling, “lmtest” (v.0.9-38) and “sandwich” (v.3.0-0) for modified Poisson regression, and “gtsummary” (v.1.4.0) for data presentation.

## Results

### Study participant characteristics

Among 284 people susceptible to HBV infection receiving free HBV vaccination coupons, 281 were eligible for analysis (98.9%). Table 1 presents the characteristics of the participants in terms of demographics, socioeconomic statuses, and social and behavioral factors. Overall, most patients were less than 40 years old, with 35.2% within the 18-30 age group. More women than men received coupons. Forty-two percent finished secondary school or lower, whereas 92% had a monthly income below $307.00 USD (roughly 7 million Viet Nam Dong as of 2022). Additionally, 43.8% of the study participants lived more than 8 kilometers from MMC. Living far from the vaccination site was significantly associated with a lower proportion of 1^st^ dose initiation.

**Table 1:** Demographics and socioeconomic statuses of participants in the total sample and stratified by the 1st dose HBV vaccine initiation

Characteristics	Total, n (column %)	Initiation of the 1^**st**^ dose		***p***-value^**b**^
		No, n (column %)	Yes, n (column %)	
**Total**	281 (100)	248 (100)	33 (100)	
**Age groups (year)**				0.218
18-30	99 (35.2)	88 (35.5)	11 (33.3)	
31-40	51 (18.1)	44 (17.7)	7 (21.2)	
41-50	59 (21.0)	56 (22.6)	3 (9.1)	
>50	72 (25.6)	60 (24.2)	12 (36.4)	
**Sex**				1.000
Female	204 (72.6)	180 (72.6)	24 (72.7)	
Male	77 (27.4)	68 (27.4)	9 (27.3)	
**Ethnicity**				1.000
Kinh	268 (96.1)	236 (95.9)	32 (97.0)	
Others	11 (3.9)	10 (4.1)	1 (3.0)	
(Missing)	2	2	0	
**Marital statuses**				0.400
Single/Separated/Divorced/Widowed	99 (35.2)	91 (38.1)	8 (26.7)	
Living together/Married	168 (64.8)	146 (61.1)	22 (73.3)	
(Missing)	14	11	3	
**Education**				0.712
No formal education	41 (14.6)	38 (15.4)	3 (9.1)	
Elementary graduate	30 (10.7)	27 (10.9)	3 (9.1)	
Secondary graduate	44 (15.7)	40 (16.2)	4 (12.1)	
High school graduate	101 (36.1)	86 (34.8)	15 (45.5)	
Undergraduate/Graduate/Postgraduate	64 (22.9)	56 (22.7)	8 (24.2)	
(Missing)	1	1	0	
**Income** ^**a**^				0.790
No Income	83 (34.7)	73 (34.3)	10 (38.5)	
< 110 USD/month	62 (25.9)	56 (26.3)	6 (23.1)	
110 to <308 USD/month	74 (31.0)	65 (30.5)	9 (34.6)	
≥ 308 USD/month	20 (8.4)	19 (8.9)	1 (3.8)	
(Missing)	42	35	7	
**Distance to the vaccination site**				0.016
8km and over	123 (43.8)	115 (46.4)	8 (24.2)	
Below 8km	158 (56.2)	133 (53.6)	25 (75.8)	
**Knowledge of Transmission**				0.009
Inadequate/0 correct	90 (32.0)	82 (33.1)	8 (24.2)	
Partial/1-4 correct	151 (53.7)	137 (55.2)	14 (42.4)	
Adequate/5-7 correct	40 (14.2)	29 (11.7)	11 (33.3)	
**Knowledge of Severity**				<0.001
Inadequate/0 correct	138 (49.1)	128 (51.6)	10 (30.3)	
Partial/1-2 correct	128 (45.6)	113 (45.6)	15 (45.5)	
Adequate/3-4 correct	15 (5.3)	7 (2.8)	8 (24.2)	
**Knowledge of HBV Vaccination**				0.600
Inadequate/0 correct	71 (25.3)	65 (26.2)	6 (18.2)	
Partial/1-2 correct	114 (40.6)	100 (40.3)	14 (42.4)	
Adequate/3-4 correct	96 (34.2)	83 (33.5)	13 (39.4)	
**Health check in the past 12 months**				0.100
No	154 (57.2)	105 (44.5)	10 (30.3)	
Don't know	39 (14.5)	36 (15.3)	3 (9.1)	
Yes	115 (42.8)	95 (40.3)	20 (60.6)	
(Missing)	12	12	0	
**Personal history of HBV vaccination**				1.000
No	219 (83.3)	192 (82.8)	27 (87.1)	
Don't know	29 (11.0)	26 (11.2)	3 (9.7)	
Yes	15 (5.7)	14 (6)	1 (3.2)	
(Missing)	18	16	2	
**Family’s history of viral hepatitis**				0.200
No	156 (58.4)	134 (56.8)	22 (71.0)	
Don't know	97 (36.3)	90 (38.1)	7 (22.6)	
Yes	14 (5.2)	12 (5.1)	2 (6.5)	
(Missing)	14	12	2	

^a^based on VND/USD conversion rates as of 2022; ^b^Chi-squared test or Fisher’s exact test where applicable

Distributions of and associations of knowledge and social and behavioral factors with the initiation of the 1^st^ dose of HBV vaccine were also presented in Table 1. 32%, 49.1%, and 25.3% of the participants had inadequate knowledge (scored 0) of transmission, severity, and HBV vaccination, respectively. 42.8% had visited doctors for health check-up(s) in the past 12 months. Among 15 participants who reported receiving HBV vaccination before study entry, only one (6.7%) initiated the first dose of the HBV vaccination series, whereas 12.1% of those who reported “No/Don’t know” on the HBV vaccination history began the first dose.

### HBV vaccine uptake and associated factors with HBV vaccine uptake

**Fig. 3:**
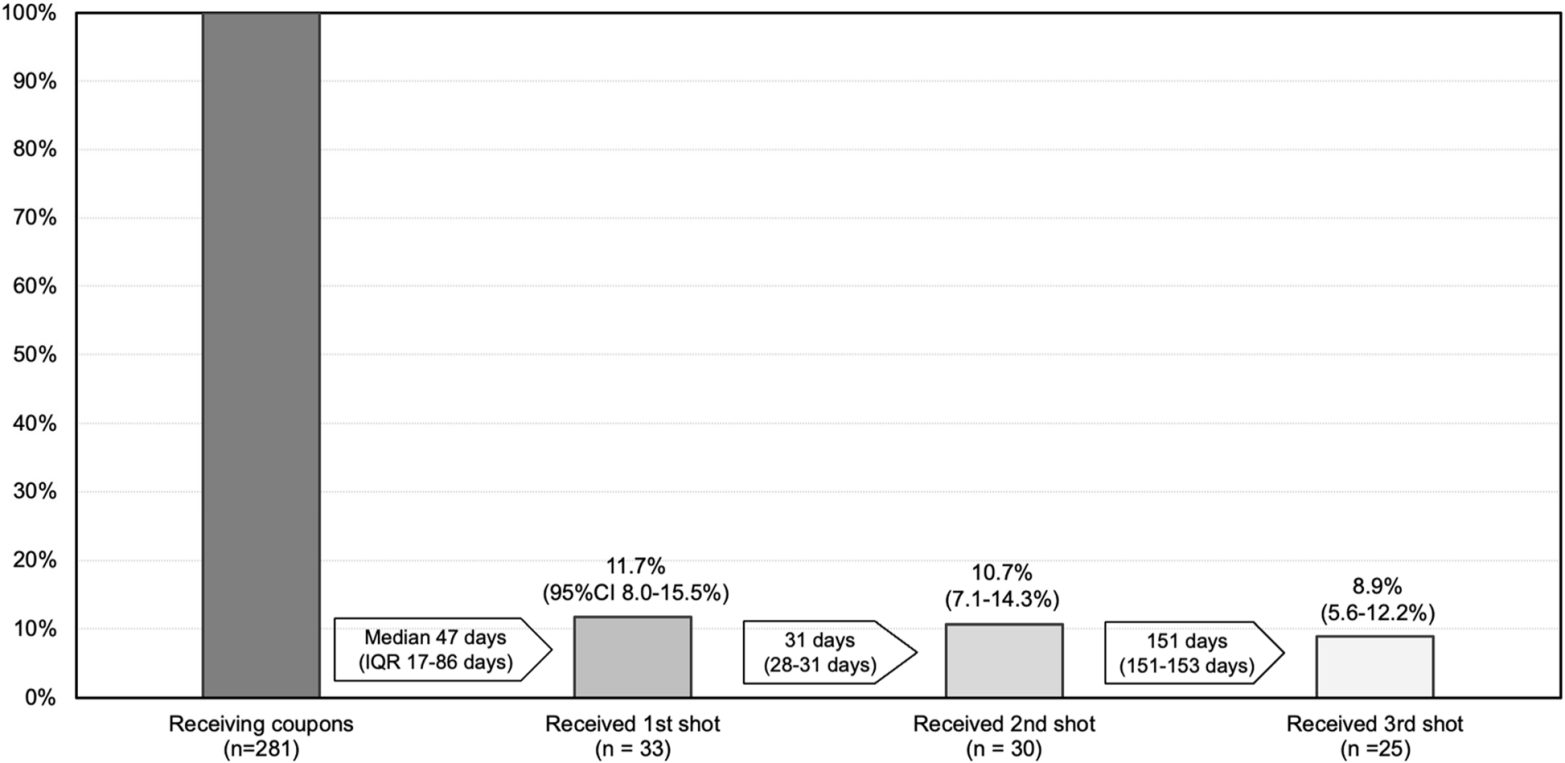
The cascade of vaccine uptake from receiving coupons to obtaining the 3rd shot. The number of those receiving coupons at baselines (the darkest bar) serves a denominator of the proportion of receiving 1st shot, 2nd shot, and 3rd shot. At the top of the other bars sit the proportions and their 95% confidence intervals of receiving each vaccine shot. The median numbers of days and their interquartile ranges between two occurrences of vaccination sit between the bars. Abbreviation: 95%CI - 95% confidence interval, IQR – Interquartile range

The cascade of vaccine uptake is shown in Figure 3. Of the 281 participants, 11.7% (95% Confidence Interval [95%CI] 8.0-15.5%) received their 1^st^ HBV vaccine dose after a median of 47 days (interquartile range [IQR] 17-86 days) upon receipt of screening results. Also, 8.9% (95%CI 5.6-12.2%) achieved series completion since the 2^nd^ dose after a median of 151 days (IQR 151-153 days), which accounted for 76.1% (25 of 33) of those who initiated the first dose.

Table 2 presents the relationship between the knowledge and socio-behavioral factors with the initiation of the first dose of the HBV vaccination. After modeling adjustments, people with adequate knowledge of HBV transmission and adequate knowledge of HBV severity were 2.58 times (95%CI 1.12-5.92) and 6.75 times (95%CI 3.38-13.48) more likely to initiate the 1^st^ dose of HBV vaccination. Also, the adjusted relative risk of 1^st^ dose initiation was 2.04 (95%CI 1.07-3.87) for those who had their health checked in the past year. No significant associations were observed for knowledge of HBV vaccination, personal HBV vaccination history, and family history of HBV. Cronbach’s alpha of three sets of knowledge questions were presented in the Appendix Table 3. Also, associations for each question item on knowledge were shown in the Appendix Table 4.

**Table 2:** Associations of knowledge and social and behavioral factors with the initiation of the 1st dose of the HBV vaccine

Characteristics	RR	95% CI	aRR	95% CI
Knowledge of transmission				
Inadequate/0 correct	—	—		
Partial/1–4 correct	1.04	0.46–2.39	0.94	0.41–2.17
Adequate/5–7 correct	3.09	1.35–7.10	2.58	1.12–5.92
**Knowledge of severity**				
Inadequate/0 correct	—	—		
Partial/1–2 correct	1.62	0.75–3.47	1.40	0.65–3.04
Adequate/3–4 correct	7.36	3.44–15.77	6.75	3.38–13.48
**Knowledge of vaccine**				
Inadequate/0 correct	—	—		
Partial/1–2 correct	1.45	0.59–3.61	1.32	0.54–3.24
Adequate/3–4 correct	1.60	0.64–4.01	1.43	0.56–3.64
**Health check in the past 12 months**				
No	—	—	—	—
Don’t know	0.88	0.26–3.05	1.06	0.32–3.52
Yes	2.00	0.98–4.08	2.06	1.02–4.20
**Personal history of HBV vaccination**				
No	—	—	—	—
Don’t know	0.84	0.27–2.59	0.81	0.25–2.67
Yes	0.54	0.08–3.71	0.55	0.08–3.73
**Family’s history of viral hepatitis**				
No	—	—	—	—
Don’t know	0.51	0.23–1.15	0.55	0.25–1.24
Yes	1.01	0.27–3.87	0.95	0.25–3.62

*Abbreviation*: *RR *Relative risk, *aRR *Relative risk adjusted for age, sex, and distance to the vaccination site, *95%CI *95% Confidence Interval

## Discussion

In this study, we found two significant findings. Firstly, one year after receiving the screening results and coupons for free vaccination, the proportion of study participants obtaining the 1^st^, 2^nd^, and 3^rd^ dose of HBV vaccine were 11.7% (95%CI 8.0-15.5%), 10.7% (95%CI 7.1-14.3%), and 8.9% (95%CI 5.6-12.2%), respectively. Secondly, knowledge of HBV transmission and severity and health-checking seeking behaviors in the past year were significantly associated with initiating the 1^st^ dose of the HBV vaccination series. To our knowledge, this study was the first in Viet Nam to distribute free vaccine coupons to HBV naïve adults born before the national EPI implementation in 2003 and follow them up to determine the vaccination uptake and its associating factors.

Three-quarters of those who initiated the first dose continued to complete the series of three doses. Although participants were provided free screening tests and vaccination coupons, the proportion of first-dose vaccine uptake was low compared with previous studies. For example, a seroprevalence survey in Ho Chi Minh City during 2019-2020 found evidence of serological HBV vaccination (i.e., isolated anti-HBs >10IU/mL) of 18.7% in the general adult population (18 years or older) [10]. Two studies on American (high-risk) [21] and French (high- and low-risk) [22] HBV naïve populations found similar proportions of initiation self-paid HBV vaccination months after free screening, with 10.6% and 11.0%, respectively. Furthermore, a clustered randomized trial on French persons at high risk for HIV observed an increase in HBV vaccine 1^st^ dose initiation from 14% to 75.6% after free testing and vaccine offers [23].

Several potential reasons may explain the low proportions of HBV vaccine uptake observed in our study. First, adult HBV vaccination is only recommended for high-risk populations (e.g., IV drug users, health care workers, sex workers) by Viet Nam’s Ministry of Health [9], while our study encouraged general adults, who are considered as low or average-risk profiles, to get vaccinated by providing free vaccination coupons. Therefore, the participants might have taken the free vaccine coupons offered less seriously. Secondly, study participants’ low HBV knowledge was also associated with low HBV vaccine uptake. A French study attributed the low proportion of 1st dose uptake to inadequate HBV knowledge because the follow-up interviews showed that common reasons were being unreceptive to vaccination or not being perceived as at-risk [22]. In our study, 25-32% of the participants had inadequate knowledge regarding HBV transmission, severity, and vaccination. Lastly, other logistical reasons, such as geographic proximity to vaccination sites, time constraints, and lack of transportation, may have also impacted vaccine uptake [14]. We observed a lower proportion of participants receiving the HBV vaccine if they lived more than 8 km from the vaccination site at the MMC.

Although HBV knowledge was conceptually associated with HBV preventive behaviors (e.g., testing, vaccine uptake) [24], the number of population-based studies on this association remains limited. Some studies, mostly adopting cross-sectional design, provided indirect supporting evidence in other populations [25, 26]. Liu et al. found that migrant workers from rural China were significantly more likely to receive the HBV vaccine if they believed that the probability of HBV exposure was high (Odds Ratio [OR] = 1.40) or that the HBV vaccine was efficient (OR = 1.21) [25]. A pooled analysis of 10 studies (mainly from the U.S.) on men who have sex with men (MSM) showed that better knowledge and perception about HBV and HBV vaccination was positively associated with increased HBV vaccination [26]. Importantly, the associations still held when we offered free HBV vaccine coupons to HBV naïve persons who just knew their HBV status through free HBV testing and followed them up.

Strategies aimed at promoting HBV vaccination in adults should appreciate the interplay of various factors at individual, provider, and policy levels [20]. In our study, even though free screening and vaccination were provided, only a small proportion of participants initiated their 1^st^ dose of vaccination. Other individual-level factors, including knowledge, beliefs, and social norms, must be considered in vaccination promotion efforts [20]. For example, educational interventions have demonstrated effectiveness in improving HBV knowledge and facilitating vaccination uptake [27, 28]. Additionally, healthcare workers (HCWs) play a crucial role in influencing vaccination decisions among their clients. Launay et al. found that a combination of free vaccination and HCWs training led to a significantly higher proportion of HBV vaccination initiation than free HBV vaccination alone in a sample of HBV-seronegative adults at increased risk for HBV infection [23]. Moreover, policy- and system-level barriers can impact both individual and provider factors. Shifting from targeted to universal recommendations for HBV vaccination is expected to have a substantial impact on vaccination uptake [12].

Limitations of the study include the following. Firstly, our study participants may not be representative of the HCMC population or other regions in Viet Nam. Secondly, some participants might have obtained HBV vaccination independently through other vaccination sites, which the study team could not track due to the unavailability of synchronized health data systems and not contacting patients directly to obtain immunization information. As a result, the proportion of vaccination might have been slightly underestimated. Additionally, while the question sets on transmission and vaccination knowledge had acceptable reliability, the set for severity knowledge was lower than the acceptable range. Hence, the association of severity knowledge on vaccination may be under- or over-estimated. Finally, future studies should expand from patients’ education to other determinants of different levels (individual, providers, system, and society) and behavioral aspects (individual capability, opportunity, and motivation) to characterize the strongest factors for effective targeted intervention.

## Conclusion

To achieve national and global HBV elimination, HBV vaccination in adult populations remains a significant gap to be addressed. Childhood HBV vaccination has been demonstrated as a cost-effective strategy worldwide and Viet Nam is one of the first countries that have been successfully implementing a national EPI for childhood HBV vaccination since 2003. We previously reported that almost 40% of the adult population in HCMC was yet protected against HBV infection. This study documented factors associated with low adulthood HBV vaccination uptake, including inadequate HBV knowledge. In 2022, the US CDC expanded its recommendation of HBV vaccination to include adults in the general population ages 19-59. This was considered an essential step toward HBV elimination in the country and globally. While different countries have different HBV epidemiologic situations, socioeconomic statuses, and healthcare priorities, our study findings are worth considering in the dialogues for national policy development to include the general adult population in Viet Nam and other countries with similar HBV epidemiological profiles.

### Supplementary Information

**Supplementary Material 1.**

## Data Availability

Individual participant data that underlie the results reported in this article, after de-identification (text, tables, figures, and appendices), will be available to researchers who provide a methodologically sound proposal. Proposals should be directed to Doan Y Dao (ddoa1@jhmi.edu) to gain access.

## Abbreviations

HBV: Hepatitis B Virus
LMIC: Lower-middle-income country
US: United States
EPI: Expanded Program on Immunization
WHO: World Health Organization
HCMC: Ho Chi Minh City
CDC: Centers for Disease Control and Prevention
CHIME: Conquering Hepatitis vIa Hepatitis Elimination
KAP: Knowledge Attitude and Practice
MMC: Medic Medical Center
SES: Socioeconomic status
OR: Odds Ratio
RR: Risk Ratio
HCW: Health Care Worker

## References

[1] Beasley RP, Hwang LY, Lin CC, Chien CS (1981). Hepatocellular carcinoma and hepatitis B virus. A prospective study of 22 707 men in Taiwan. Lancet.

[2] Global progress report on HIV, viral hepatitis and sexually transmitted infections, 2021. Accountability for the global health sector strategies 2016–2021: actions for impact. Geneva: World Health Organization; 2021. License: CC BY-NC-SA 3.0 IGO.

[3] Alter MJ (2003). Epidemiology of hepatitis B in Europe and worldwide. J Hepatol.

[4] Pham TND, Le DH, Dao DVB, Phan LTB, Pham TTT, Nguyen TB (2023). Establishing baseline framework for hepatitis B virus micro-elimination in Ho Chi Minh City, Vietnam - a community-based seroprevalence study. Lancet Reg Health West Pac.

[5] Pham T, Bui L, Kim G, Hoang D, Tran T, Hoang M (2019). Cancers in Vietnam-burden and control efforts: a narrative scoping review. Cancer Control.

[6] Nguyen-Dinh SH, Do A, Pham TND, Dao DY, Nguy TN, Chen MS (2018). High burden of hepatocellular carcinoma and viral hepatitis in Southern and Central Vietnam: experience of a large tertiary referral center, 2010 to 2016. World J Hepatol.

[7] Komada K, Ichimura Y, Shimada M, Funato M, Do HT, Le HX (2022). Impact of hepatitis B vaccination programs in Vietnam evaluated by estimating HBsAg prevalence. J Virus Erad.

[8] Global Hepatitis Report 2017. Geneva: World Health Organization; 2017. License: CC BY-NC-SA 3.0 IGO.

[9] National Action Plan for Prevention of Viral Hepatitis in 2021–2025. Ha Noi: Viet Nam Ministry of Health; 2021.

[10] Kim TV, Pham TND, Le DH, Dao DVB, Phan LTB, Le A (2023). Significant gaps in hepatitis B vaccination in adults in Viet Nam: important targets toward hepatitis B elimination by 2030. Vaccine.

[11] Global health sector strategy on viral hepatitis 2016–2021. Towards ending viral hepatitis. Geneva: World Health Organization; 2016. License: CC BY-NC-SA 3.0 IGO.

[12] Weng MK, Doshani M, Khan MA, Frey S, Ault K, Moore KL (2022). Universal Hepatitis B vaccination in adults aged 19–59 years: updated recommendations of the advisory committee on immunization practices—United States, 2022. Morb Mortal Wkly Rep.

[13] Kim TV, Le DH, Dao DVB, Pham TND, Mize GW, Phan LTB (2022). Demonstration of a population-based HCV serosurvey in Ho Chi Minh City, Viet Nam: establishing baseline prevalence of and continuum of care for HCV micro-elimination by 2030. Lancet Reg Health West Pac.

[14] Nguyen T, Pham T, Phan L, Mize G, Trang A, Dao D (2021). Progressive scale-up of HBV AND HCV testing for hepatitis elimination in Vietnam. Clin Liver Dis.

[15] La Roche Ltd. Elecsys® anti-HBs II: immunoassay for the quantitative determination of antibodies to hepatitis B surface antigen (HBsAg). Available from: https://diagnostics.roche.com/global/en/products/params/elecsys-anti-hbs-ii.html.

[16] La Roche Ltd. Elecsys® anti-HBc II: immunoassay for the qualitative determination of total antibodies against hepatitis B core antigen (HBcAg). Available from: https://diagnostics.roche.com/global/en/products/params/elecsys-anti-hbc-ii.html.

[17] La Roche Ltd. Elecsys® HBsAg II: immunoassay for the qualitative determination of hepatitis B surface antigen (HBsAg). Available from: https://diagnostics.roche.com/global/en/products/params/elecsys-hbsag-ii.html.

[18] Maxwell AE, Bastani R, Chen MS, Nguyen TT, Stewart SL, Taylor VM (2010). Constructing a theoretically based set of measures for liver cancer control research studies. Prev Med.

[19] Zou G (2004). A modified poisson regression approach to prospective studies with binary data. Am J Epidemiol.

[20] Bastani R, Glenn BA, Taylor VM, Chen MS, Nguyen TT, Stewart SL (2010). Integrating theory into community interventions to reduce liver cancer disparities: the health behavior framework. Prev Med.

[21] Hechter RC, Jacobsen SJ, Luo Y, Nomura JH, Towner WJ, Tartof SY (2014). Hepatitis B testing and vaccination among adults with sexually transmitted infections in a large managed care organization. Clin Infect Dis.

[22] Boyd A, Bottero J, Carrat F, Gozlan J, Rougier H, Girard PM (2017). Testing for hepatitis B virus alone does not increase vaccine coverage in non-immunized persons. World J Gastroenterol.

[23] Launay O, Le Strat Y, Tosini W, Kara L, Quelet S, Lévy S (2014). Impact of free on-site vaccine and/or healthcare workers training on hepatitis B vaccination acceptability in high-risk subjects: a pre-post cluster randomized study. Clin Microbiol Infect.

[24] Bastani R, Glenn BA, Taylor VM, Chen MS, Nguyen TT, Stewart SL (2010). Integrating theory into community interventions to reduce liver cancer disparities: the health behavior framework. Prev Med.

[25] Liu R, Li Y, Wangen KR, Maitland E, Nicholas S, Wang J (2016). Analysis of hepatitis B vaccination behavior and vaccination willingness among migrant workers from rural China based on protection motivation theory. Hum Vaccin Immunother.

[26] Vet R, de Wit JB, Das E (2017). Factors associated with hepatitis B vaccination among men who have sex with men: a systematic review of published research. Int J STD AIDS.

[27] Zacharias T, Wang W, Dao D, Wojciechowski H, Lee WM, Do S (2015). HBV outreach programs significantly increase knowledge and vaccination rates among Asian pacific islanders. J Community Health.

[28] Shah HA, Abu-Amara M (2013). Education provides significant benefits to patients with hepatitis B virus or hepatitis C virus infection: a systematic review. Clin Gastroenterol Hepatol.

