# Supplementary material for: Low HBV knowledge is associated with low HBV vaccination uptake in general adult population despite incentivization of HBV vaccination
Source: BMC Infect Dis. 2024 May 3;24:470. doi: 10.1186/s12879-024-09326-9 (PMC11067299; doi:10.1186/s12879-024-09326-9)
Supplement: Supplementary file 1 — Supplementary Material 1. [file 12879_2024_9326_MOESM1_ESM.docx]

# **SUPPLEMENTAL APPENDIX**

***Appendix Table 1*** *Comparison of characteristics between the population in the current study (HBV naïve) and other populations with different HBV statuses*

| **Characteristic** | **Total** | **HBV status** | | | | | **p-value^b^** |
| --- | --- | --- | --- | --- | --- | --- | --- |
|  |  | **HBV Naïve**  **n (column %)** | **Vaccinated**  **n (column %)** | **Recovered**  **n (column %)** | **Infected**  **n (column %)** | **Isolated  anti-HBc Ab**  **n (column %)** |  |
| **Total** | **1,008** | **284** | **146** | **380** | **72** | **126** |  |
| **Age groups (year)** |  |  |  |  |  |  | <0.001 |
| 18-30 | 211.0 (20.9%) | 99.0 (34.9%) | 41.0 (28.1%) | 50.0 (13.2%) | 14.0 (19.4%) | 7.0 (5.6%) |  |
| 31-40 | 174.0 (17.3%) | 52.0 (18.3%) | 28.0 (19.2%) | 56.0 (14.7%) | 15.0 (20.8%) | 23.0 (18.3%) |  |
| 41-50 | 240.0 (23.8%) | 61.0 (21.5%) | 33.0 (22.6%) | 96.0 (25.3%) | 23.0 (31.9%) | 27.0 (21.4%) |  |
| >50 | 383.0 (38.0%) | 72.0 (25.4%) | 44.0 (30.1%) | 178.0 (46.8%) | 20.0 (27.8%) | 69.0 (54.8%) |  |
| **Sex** |  |  |  |  |  |  | **0.007** |
| Female | 684.0 (67.9%) | 206.0 (72.5%) | 101.0 (69.2%) | 262.0 (68.9%) | 37.0 (51.4%) | 78.0 (61.9%) |  |
| Male | 324.0 (32.1%) | 78.0 (27.5%) | 45.0 (30.8%) | 118.0 (31.1%) | 35.0 (48.6%) | 48.0 (38.1%) |  |
| **Ethnicity** |  |  |  |  |  |  | 0.3 |
| Kinh | 949.0 (95.8%) | 271.0 (96.1%) | 134.0 (92.4%) | 358.0 (96.8%) | 69.0 (97.2%) | 117.0 (95.1%) |  |
| Others | 42.0 (4.2%) | 11.0 (3.9%) | 11.0 (7.6%) | 12.0 (3.2%) | 2.0 (2.8%) | 6.0 (4.9%) |  |
| (Missing) | 17 | 2 | 1 | 10 | 1 | 3 |  |
| **Marital statuses** |  |  |  |  |  |  | **0.007** |
| Single/Separated/Divorced/Widowed | 304.0 (31.0%) | 99.0 (36.4%) | 46.0 (31.7%) | 111.0 (29.5%) | 16.0 (23.5%) | 32.0 (26.4%) |  |
| Living together/Married | 670.0 (68.2%) | 171.0 (62.9%) | 97.0 (66.9%) | 264.0 (70.2%) | 49.0 (72.1%) | 89.0 (73.6%) |  |
| (Missing) | 34 | 14 | 3 | 5 | 7 | 5 |  |
| **Education** |  |  |  |  |  |  | **<0.001** |
| No formal education | 164.0 (16.3%) | 41.0 (14.5%) | 14.0 (9.6%) | 69.0 (18.2%) | 17.0 (23.9%) | 23.0 (18.3%) |  |
| Elementary graduate | 112.0 (11.1%) | 31.0 (11.0%) | 10.0 (6.8%) | 46.0 (12.1%) | 5.0 (7.0%) | 20.0 (15.9%) |  |
| Secondary graduate | 223.0 (22.2%) | 44.0 (15.5%) | 29.0 (19.9%) | 95.0 (25.1%) | 20.0 (28.2%) | 35.0 (27.8%) |  |
| High school graduate | 304.0 (30.2%) | 103.0 (36.4%) | 48.0 (32.9%) | 108.0 (28.5%) | 15.0 (21.1%) | 30.0 (23.8%) |  |
| Undergraduate/Graduate/Postgraduate | 202.0 (20.1%) | 64.0 (22.6%) | 45.0 (30.8%) | 61.0 (16.1%) | 14.0 (19.7%) | 18.0 (14.3%) |  |
| (Missing) | 3 | 1 | 0 | 1 | 1 | 0 |  |
| **Income^a^** |  |  |  |  |  |  | 0.785 |
| No Income | 309.0 (36.1%) | 84.0 (34.9%) | 39.0 (30.2%) | 125.0 (38.8%) | 21.0 (40.4%) | 40.0 (36.0%) |  |
| < 110 USD/month | 199.0 (23.3%) | 62.0 (25.7%) | 28.0 (21.7%) | 76.0 (23.6%) | 13.0 (25.0%) | 20.0 (18.0%) |  |
| 110 to <308 USD/month | 273.0 (31.9%) | 75.0 (31.1%) | 49.0 (38.0%) | 97.0 (30.1%) | 14.0 (26.9%) | 38.0 (34.2%) |  |
| 308 and higher USD/month | 74.0 (8.7%) | 20.0 (8.3%) | 13.0 (10.1%) | 24.0 (7.5%) | 4.0 (7.7%) | 13.0 (11.7%) |  |
| (Missing) | 153 | 43 | 17 | 58 | 20 | 15 |  |
| **Distance to the vaccination site** |  |  |  |  |  |  | 0.3 |
| 8km and over | 451.0 (44.7%) | 123.0 (43.3%) | 64.0 (43.8%) | 176.0 (46.3%) | 39.0 (54.2%) | 49.0 (38.9%) |  |
| Below 8km | 557.0 (55.3%) | 161.0 (56.7%) | 82.0 (56.2%) | 204.0 (53.7%) | 33.0 (45.8%) | 77.0 (61.1%) |  |
| **Knowledge of Transmission** |  |  |  |  |  |  | **0.011** |
| Inadequate/0 correct | 319.0 (31.7%) | 91.0 (32.0%) | 32.0 (21.9%) | 133.0 (35.1%) | 20.0 (28.2%) | 43.0 (34.1%) |  |
| Partial/1-4 correct | 515.0 (51.2%) | 152.0 (53.5%) | 73.0 (50.0%) | 190.0 (50.1%) | 37.0 (52.1%) | 63.0 (50.0%) |  |
| Adequate/5-7 correct | 172.0 (17.1%) | 41.0 (14.4%) | 41.0 (28.1%) | 56.0 (14.8%) | 14.0 (19.7%) | 20.0 (15.9%) |  |
| (Missing) | 2 | 0 | 0 | 1 | 1 | 0 |  |
| **Knowledge of Severity** |  |  |  |  |  |  |  |
| Inadequate/0 correct | 449.0 (44.6%) | 139.0 (48.9%) | 54.0 (37.0%) | 181.0 (47.8%) | 26.0 (36.6%) | 49.0 (38.9%) | 0.176 |
| Partial/1-2 correct | 490.0 (48.7%) | 130.0 (45.8%) | 81.0 (55.5%) | 170.0 (44.9%) | 41.0 (57.7%) | 68.0 (54.0%) |  |
| Adequate/3-4 correct | 67.0 (6.7%) | 15.0 (5.3%) | 11.0 (7.5%) | 28.0 (7.4%) | 4.0 (5.6%) | 9.0 (7.1%) |  |
| (Missing) | 2 | 0 | 0 | 1 | 1 | 0 |  |
| **Knowledge of HBV Vaccination** |  |  |  |  |  |  | **0.036** |
| Inadequate/0 correct | 210.0 (20.8%) | 72.0 (25.4%) | 24.0 (16.4%) | 78.0 (20.5%) | 11.0 (15.3%) | 25.0 (19.8%) |  |
| Partial/1-2 correct | 395.0 (39.2%) | 115.0 (40.5%) | 47.0 (32.2%) | 156.0 (41.1%) | 32.0 (44.4%) | 45.0 (35.7%) |  |
| Adequate/3-4 correct | 403.0 (40.0%) | 97.0 (34.2%) | 75.0 (51.4%) | 146.0 (38.4%) | 29.0 (40.3%) | 56.0 (44.4%) |  |
| **Health check in the past 12 months** |  |  |  |  |  |  | **0.051** |
| No/Don't know | 501.0 (51.9%) | 157.0 (57.7%) | 71.0 (50.0%) | 188.0 (52.2%) | 28.0 (39.4%) | 57.0 (47.1%) |  |
| Yes | 465.0 (48.1%) | 115.0 (42.3%) | 71.0 (50.0%) | 172.0 (47.8%) | 43.0 (60.6%) | 64.0 (52.9%) |  |
| (Missing) | 42 | 12 | 4 | 20 | 1 | 5 |  |
| **Personal history of HBV vaccination** |  |  |  |  |  |  | **<0.001** |
| No/Don't know | 854.0 (89.8%) | 251.0 (94.4%) | 98.0 (70.0%) | 326.0 (90.8%) | 65.0 (95.6%) | 114.0 (96.6%) |  |
| Yes | 97.0 (10.2%) | 15.0 (5.6%) | 42.0 (30.0%) | 33.0 (9.2%) | 3.0 (4.4%) | 4.0 (3.4%) |  |
| (Missing) | 57 | 18 | 6 | 21 | 4 | 8 |  |
| **Family’s history of viral hepatitis** |  |  |  |  |  |  | **0.011** |
| No/Don't know | 870.0 (91.1%) | 256.0 (94.8%) | 128.0 (91.4%) | 316.0 (89.0%) | 58.0 (82.9%) | 112.0 (93.3%) |  |
| Yes | 85.0 (8.9%) | 14.0 (5.2%) | 12.0 (8.6%) | 39.0 (11.0%) | 12.0 (17.1%) | 8.0 (6.7%) |  |
| (Missing) | 53 | 14 | 6 | 25 | 2 | 6 |  |
| ^a^based on VND/USD conversion rates as of 2022; ^b^Chi-squared test or Fisher’s exact test;  Note: The shaded column represents the population of interest in the current study; HBV naïve defined as HBsAg (-), anti-HBs Ab< 10mIU/mL, and anti-HBc total Ab (-); Vaccinated defined as HBsAg (-), anti-HBs Ab ≥ 10 mIU/mL, anti-HBc total Ab (-); Recovered defined as HBsAg (-), anti-HBs Ab ≥ 10mIU/mL, and anti-HBc total Ab (+); Infected defined as isolated HBsAg (+) | | | | | | | |
|  | | | | | | | |
|  | | | | | | | |

***Appendix Table 2*** *Distance from participants’ residential address to Medic Medical Center (Shortest route on Google Map from the geographical midpoint of the residential ward to Medic Medical Center as of March 2023)*

| **Residence** | **Distance (km)** |
| --- | --- |
| Ward 5, District 8 | 4.6 |
| Ward 3, District Binh Thanh | 5.4 |
| Ward 10, District 6 | 7.1 |
| Ward 9, District Phu Nhuan | 7.7 |
| Ward Son Ky, District Tan Phu | 9.6 |
| Ward 5, District Go Vap | 10.0 |
| Ward 22, District Binh Thanh | 11.2 |

***Appendix Table 3*** *Internal reliability of three sets of questions*

| **Question sets** | **Cronbach's alpha** | **95%CI** |
| --- | --- | --- |
| Knowledge of transmission (7 items) | 0.756 | 0.704-0.799 |
| Knowledge of severity (4 items) | 0.51 | 0.380-0.608 |
| Knowledge of vaccination (4 items) | 0.665 | 0.599-0.724 |

***Appendix Table 4*** *Associations of each question item on knowledge and social and behavioral factors with the initiation of the 1^st^ dose of the HBV vaccine*

| **Characteristics** | **Total** | **1^st^ dose initiation** | **RR** | **95% CI** | **aRR^3^** | **95% CI** |
| --- | --- | --- | --- | --- | --- | --- |
| **Knowledge of transmission** | | |  |  |  |  |
| **Individual questions** |  |  |  |  |  |  |
| Smoking | 81 (29%) | 10 (30%) | 1.07 | 0.54-2.15 | 1.05 | 0.52-2.11 |
| Sharing food | 76 (27%) | 8 (24%) | 0.86 | 0.41-1.83 | 0.92 | 0.43-1.93 |
| Brushing tooth | 101 (36%) | 16 (48%) | 1.67 | 0.88-3.16 | 1.43 | 0.74-2.73 |
| Sneezing | 70 (25%) | 11 (33%) | 1.51 | 0.77-2.95 | 1.44 | 0.73-2.85 |
| Commercial sex | 98 (35%) | 18 (55%) | **2.24** | **1.18-4.25** | **2.05** | **1.10-3.82** |
| Sharing needles | 117 (42%) | 21 (64%) | **2.45** | **1.26-4.79** | **2.13** | **1.13-4.03** |
| Giving birth | 119 (42%) | 18 (55%) | 1.63 | 0.86-3.11 | 1.43 | 0.76-2.70 |
| **Knowledge of severity** | | |  |  |  |  |
| **Individual questions** |  |  |  |  |  |  |
| Life long infection | 10 (3.6%) | 0 (0%) | 0.00 | 0.00-Inf | 0.00 | 0.00-0.00 |
| Liver cancer | 116 (41%) | 18 (55%) | 1.71 | 0.90-3.25 | 1.47 | 0.77-2.81 |
| Death | 108 (38%) | 20 (61%) | **2.46** | **1.28-4.75** | **2.21** | **1.14-4.28** |
| Being treatable | 19 (6.8%) | 9 (27%) | **5.17** | **2.81-9.50** | **4.95** | **2.78-8.80** |
| **Knowledge of vaccine** | | |  |  |  |  |
| **Individual questions** |  |  |  |  |  |  |
| Effectiveness | 159 (57%) | 21 (64%) | 1.34 | 0.69-2.62 | 1.33 | 0.69-2.57 |
| Safety | 162 (58%) | 22 (67%) | 1.47 | 0.74-2.91 | 1.42 | 0.73-2.74 |
| Adverse events | 77 (27%) | 14 (42%) | **1.95** | **1.03-3.70** | 1.87 | 0.98-3.56 |
| Vaccination facilities | 98 (35%) | 10 (30%) | 0.81 | 0.40-1.64 | 0.67 | 0.32-1.38 |
| *Abbreviation: RR – Relative risk, aRR – relative risk adjusted for age, sex, and distance to the vaccination site, 95%CI – 95% Confidence Interval* | | | | | | |

***Precision and Power analysis***

Regarding our 1^st^ research question of precision, we plug these numbers into the formula to estimate a proportion. We used an interventional study by Boyd et al., with the proportion of HBV vaccination of ≈10%, as a reference

$$n\geq\frac{z_{1-\propto/2}^{2}x p x (1-p)}{d^{2}} =\frac{{1.96}^{2}x 0.1 x \left( 1-0.1 \right)}{{0.05}^{2}}= 139 participants$$

Regarding the 2^nd^ question using multiple regression with sufficient power, we apply the formula suggested by Vittinghoff et al.^1^ and Hosmer et al.^2^ to ensure enough events per covariate.

$$p+1\leq\frac{number of events}{5 to 9 participants}$$

$$p\leq\frac{30}{5 to 9 participants}-1=2.3 to 5 parameters$$

Therefore, with 281 participants, 32 of whom had events (HBV vaccination), we have sufficient precision to calculate the proportion of vaccination and enough statistical power to adjust for four parameters in the multiple regression.

1. Vittinghoff, E. and McCulloch, C.E., 2007. Relaxing the rule of ten events per variable in logistic and Cox regression. *American journal of epidemiology*, *165*(6), pp.710-718.

2. Hosmer Jr, D.W., Lemeshow, S. and Sturdivant, R.X., 2013. *Applied logistic regression*. John Wiley & Sons.

## ***The questionnaire***

| ***Questions*** | ***Options*** (options in bold and underlined were coded as 1 if chosen, otherwise as 0) |
| --- | --- |
| Do you think it is possible to get viral hepatitis from smoking? | □ Yes for HBV  □ Yes for HCV  □ Yes for both HBV and HCV  **□ No**  □ Don't know |
| Do you think it is possible to get viral hepatitis from eating or drinking together or sharing  spoons, chopsticks and forks? | □ Yes for HBV  □ Yes for HCV  □ Yes for both HBV and HCV  □ **No**  □ Don't know |
| Do you think it is possible to get viral hepatitis from sharing toothbrushes? | □ **Yes for HBV**  □ **Yes for HCV**  □ **Yes for both HBV and HCV**  □ No  □ Don't know |
| Do you think it is possible to get a viral infection from being around someone who is  sneezing or coughing? | □ Yes for HBV  □ Yes for HCV  □ Yes for both HBV and HCV  □ **No**  □ Don't know |
| Do you think it is possible to get viral hepatitis from sex? | □ **Yes for HBV**  □ **Yes for HCV**  □ **Yes for both HBV and HCV**  □ No  □ Don't know |
| Do you think it is possible to get viral hepatitis from sharing or reusing needles such as acupuncture, tattooing, or injecting with used needles? | □ **Yes for HBV**  □ **Yes for HCV**  □ **Yes for both HBV and HCV**  □ No  □ Don't know |
| Do you think that the baby can get viral hepatitis due to transmission from the mother during  birth? | □ **Yes for HBV**  □ **Yes for HCV**  □ **Yes for both HBV and HCV**  □ No  □ Don't know |
| Do you think that an asymptomatic person with viral hepatitis can still transmit the hepatitis  virus? | □ **Yes for HBV**  □ **Yes for HCV**  □ **Yes for both HBV and HCV**  □ No  □ Don't know |
| Do you think that people who have been infected with viral hepatitis will be infected for life? | □ **Yes for HBV**  □ Yes for HCV  □ Yes for both HBV and HCV  □ No  Don't know |
| Do you think viral hepatitis can lead to liver cancer? | □ **Yes for HBV**  □ **Yes for HCV**  □ **Yes for both HBV and HCV**  □ No  □ Don't know |
| Do you think a person can die from viral hepatitis? | □ **Yes for HBV**  □ **Yes for HCV**  □ **Yes for both HBV and HCV**  □ No  □ Don't know |
| Do you think viral hepatitis can be cured? | □ Yes for HBV  □ **Yes for HCV**  □ Yes for both HBV and HCV  □ No  □ Don't know |
| Do you think the hepatitis B vaccine is effective in preventing hepatitis B? | □ **Yes**  □ No  □ Don't know |
| Do you believe that the hepatitis B vaccine can cause harmful side effects in many people? | □ Yes  □ **No**  □ Don't know |
| Do you believe the hepatitis B vaccine is safe? | □ **Yes**  □ No  □ Don't know |
| Do you know where you can get the hepatitis B vaccine? | □ **Yes**  □ Don't know |

-- END --
